# Polymorphisms in the xenobiotic transporter Multidrug Resistance 1 (MDR1) and interaction with meat intake in relation to risk of colorectal cancer in a Danish prospective case-cohort study

**DOI:** 10.1186/1471-2407-9-407

**Published:** 2009-11-21

**Authors:** Vibeke Andersen, Mette Østergaard, Jane Christensen, Kim Overvad, Anne Tjønneland, Ulla Vogel

**Affiliations:** 1 Medical Department, Viborg Regional Hospital, DK-8800 Viborg, Denmark; 2 Clinical Biochemical Department, Viborg Regional Hospital, DK-8800 Viborg, Denmark; 3 Danish Cancer Society, Institute of Cancer Epidemiology, DK-2100 Copenhagen, Denmark; 4 Department of Clinical Epidemiology, Aarhus University Hospital, DK-9100 Aalborg, Denmark; 5 National Research Centre for the Working Environment, DK-2100 Copenhagen, Denmark; 6 National Food Institute, Technical University of Denmark, DK-2860 Søborg, Denmark

## Abstract

**Background:**

The xenobiotic transporters, Multidrug Resistance 1 *(MDR1/ABCB1) *and Breast Cancer Resistance Protein *(BCRP/ABCG2) *may restrict intestinal absorption of various carcinogens, including heterocyclic amines (HCA) and polycyclic aromatic hydrocarbons (PAH). Cyclooxygenase-2 (COX-2) derived prostaglandins promote gastrointestinal carcinogenesis, affecting angiogenesis, apoptosis, and invasiveness.

The aim of this study was to investigate if polymorphisms in these genes were associated with risk of colorectal cancer (CRC), and to investigate possible interactions with lifestyle factors such as smoking, meat consumption, and NSAID use.

**Methods:**

The following polymorphisms were analyzed; a synonymous *MDR1 *C3435T (rs1045642) in exon26, G-rs3789243-A in intron3, the functional *BCRP *C421A (rs2231142), the two *COX-2 *A-1195G (rs689466) and G-765C (rs20417) in the promoter region, and the *COX-2 *T8473C (rs5275) polymorphisms in the 3'-untranslated region. The polymorphisms were assessed together with lifestyle factors in a nested case-cohort study of 359 cases and a random cohort sample of 765 participants from the Danish prospective Diet, Cancer and Health study.

**Results:**

Carriers of the variant allele of *MDR1 *intron 3 polymorphism were at 1.52-fold higher risk of CRC than homozygous wild type allele carriers (Incidence rate ratio (IRR) = 1.52, 95% Confidence Interval (CI): 1.12-2.06). Carriers of the variant allele of *MDR1 *C3435T exon 26 had a lower risk of CRC than homozygous C-allele carriers (IRR = 0.71 (CI:0.50-1.00)). There was interaction between these *MDR1 *polymorphisms and intake of red and processed meat in relation to CRC risk. Homozygous *MDR1 *C3435T C-allele carriers were at 8% increased risk pr 25 gram meat per day (CI: 1.00-1.16) whereas variant allele carriers were not at increased risk (p for interaction = 0.02). *COX-2 *and *BCRP *polymorphisms were not associated with CRC risk. There was interaction between NSAID use and *MDR1 *C3435T and COX-2 T8473C (p-values for interaction 0.001 and 0.04, respectively).

**Conclusion:**

Two polymorphisms in *MDR1 *were associated with CRC risk and there was interaction between these polymorphisms and meat intake in relation to CRC risk. Our results suggest that *MDR1 *polymorphisms affect the relationship between meat and CRC risk.

## Background

Colorectal cancer (CRC) is one of the leading causes of cancer-related mortality in the Western World, with great impact on the life quality for the affected persons. Both genetic and life-style factors contribute to the pathogenesis, and gene-environmental interactions may modulate cancer risk. Polymorphisms in genes encoding enzymes involved in the transport and metabolism of ingested carcinogens may affect risk of CRC. In line with this, interactions between genetic polymorphisms affecting the metabolism of dietary carcinogens and meat intake have been found in relation to CRC [1,2], whereas studies on the potential interactions between environmental exposure in terms of meat consumption and cigarette smoking on one side and genetic polymorphisms in genes affecting intestinal carcinogen transport on the other are scarce [3,4].

The ATP-binding cassette (ABC) transporters P-glycoprotein (encoded by the Multidrug Resistance 1 (*MDR1/ABCB1*) gene) and Breast Cancer Resistance Protein (BCRP, encoded by the *BCRP/ABCG2 *gene) are abundant in the intestine [5]. They transport a diverse spectrum of substrates from the enterocytes into the intestinal lumen, thereby restricting the exposure to these potentially harmful substances [6]. The substrates include a vast amount of structurally unrelated compounds such as various drugs [7,8]. Moreover, pesticides and insecticides [9], carcinogens such as PAHs [10] and HCAs [6,11,12] and endogenous compounds such as steroids and cytokines [13,14] have also been suggested as substrates. One of the most abundant dietary carcinogens formed during frying and cooking of meat, 2-Amino-1-methyl-6-phenylimidazo [4,5-b]pyridine (PhIP), is transported by BCRP [10], and probably to a lesser extent by P-glycoprotein [6]. P-glycoprotein preferentially transports large hydrophobic molecules, while BCRP is able to transport both hydrophobic and large anionic compounds, e.g. conjugates, however, the substrate specificities have been shown to be overlapping [7].

Intake of red and processed meat have been classified as risk factor for colorectal cancer (CRC) [15,16]. The exact mechanisms by which intake of meat and processed meat promotes carcinogenesis is, however, not clear but several different mechanisms have been proposed [17]. First, red and processed meat may be a proxy for a high fat diet or other life-style factors. Next, red and processed meat represent sources of carcinogenic heterocyclic amines (HCA), polycyclic aromatic hydrocarbons (PAH) as well as N-nitroso compounds [17] caused by cooking at high temperature and by processing of meat. Moreover, heme iron may promote carcinogenesis in combination with N-nitroso compounds [17]. Smoking has also been reported to confer risk of CRC [18,19] and tobacco contains a large number of mutagens and carcinogens, including PAH, nitrosamines, and nicotine [20] that may reach the intestine directly via ingestion of inhaled particles which are subsequently swallowed. PAHs and other carcinogens adhered on ingested particles may dissociate from the particles and be taken up the same way as meat carcinogens.

There is increasing evidence that polymorphisms in *MDR1 *affect P-glycoprotein activity and expression [21-23]. The synonymous *MDR1 *C3435T polymorphism in exon 26 has been most extensively investigated. The variant allele has been associated with lower *in vitro *activity and changed substrate specificity, possibly caused by a lower mRNA stability and protein folding [24,25], whereas studies on intestinal mRNA levels and protein expression levels are not consistent [22,26-28]. The potential functional effect of the *MDR1 *G-rs3789243A polymorphism in intron 3 is unknown [29,30]. *MDR1 *polymorphisms have been studied in relation to risk of CRC in previous studies, however, results are inconsistent [3,31-33].

The variant allele of the non-synonymous *BCRP *C421A (Q141K) polymorphism has been associated with lower protein levels and lowered transport activity both *in vitro *[34,35] and *in vivo *[27,35], but no associations were found between either the intestinal levels of protein and mRNA and BRCP polymorphisms [36] or between *BCRP *polymorphisms and risk of CRC [4].

Cyclooxygenase-2 (COX-2) plays a key role in gastrointestinal carcinogenesis, affecting angiogenesis, apoptosis, and invasiveness [37]. Moreover, COX-2 is involved in the regulation of the intestinal immune response to luminal antigens [38] and modulate the interaction between carcinogen exposure and intestinal barrier function by regulation of the intestinal immune homeostasis.

Two polymorphisms, A-1195G and G-765C in the promoter region of *COX-2 *have been described [39]. Carriers of the variant G-allele of -1195 lack a c-Myb binding site resulting in lowered COX-2 mRNA levels [39]. The G to C substitution at nucleotide -765 eliminates an Sp1 binding site, but meanwhile creates an E2F binding site [39]. Even though *in vitro *studies have revealed a lower COX-2 expression from the C-allele of G-765C allele [39], *in vivo *studies have shown the opposite effect [39,40]. A *COX-2 *haplotype containing the variant allele in position -765 together with the variant allele of the T8473C polymorphism in the 3' untranslated region (UTR) has been associated with high COX-2 activity [41], indicating that the 3'UTR variant allele may stabilize the mRNA level. The *COX-2 *A-1195G wildtype and G-765C variant alleles were associated with increased risk of CRC in a large Chinese study [42] in accordance with a high COX-2 level conferred by these alleles.

Long term use of aspirin and other non-steroidal anti-inflammatory drugs (NSAID) has been found to confer protection against CRC [43,44]. The mechanism is considered to be inhibition of COX-2 activity but also COX-2 independent pathways are involved which includes antigen activation of gut inflammation [45]. Smoking has been shown to induce inflammation [46] and *COX-2 *expression [47,48]. Therefore, we hypothesized that polymorphisms affecting the intestinal barrier, i.e. intestinal transporters, and intestinal immune response may modulate the effects of smoking, meat intake and use of NSAIDs in relation to CRC risk. A priori, we expected that the variant alleles of *BCRP *C421A, the *MDR1 *C3435T, *MDR1 *G-rs3789243-A, the *COX-2 *G-765C and T8473C were risk alleles, and the *COX-2 *A-1195G wildtype allele was the high risk allele. We searched for associations between polymorphisms in *MDR1, BCRP *and *COX-2 *and risk of CRC as well as the potential interaction with smoking, consumption of meat, and use of NSAID in a case-cohort study nested in the prospective population-based Danish Diet, Cancer and Health cohort study.

## Methods

### Studied Subjects

The subjects were selected from the Danish Diet, Cancer and Health study, an ongoing prospective cohort study [49]. Between December 1993 and May 1997, 160,725 individuals aged 50 to 64 years, born in Denmark, living in the Copenhagen and Aarhus areas and having no previous cancers at the time of invitation, were invited to participate in the study. A total of 57,053 persons accepted the invitation.

In total, 405 cases (184 women and 221 men) of colorectal cancer were diagnosed among the cohort members between 1994 and 2003 and registered in the files of the nationwide Danish Cancer Registry. Within the cohort we defined a sub-cohort sample including 368 women and 442 men who were randomly selected. Cases and the sub-cohort sample were frequency-matched on gender. Blood samples were available for 397 cases and 800 sub-cohort members. All information on genotypes and lifestyle factors was available for 372 cases and 765 sub-cohort members. 13 colorectal cancer cases diagnosed with carcinoid tumor or various other histological subtypes were excluded from the analysis, leaving 359 adenocarcinoma cases.

### Lifestyle variables

At enrolment, detailed information on diet, lifestyle, weight, height, medical treatment, environmental exposures, and other socio-economic factors were collected [49]. In the food-frequency questionnaire, meat consumption was assessed in 12 categories of predefined responses, ranking from 'never' to 'eight times or more per day'. The daily intake was then calculated by using FoodCalc [50], this program uses population specific standardized recipes and portion sizes. Intake of red meat in grams per day was calculated by adding up intake of beef, veal, pork, lamb and offal. Intake of processed meat in grams per day was calculated by adding up intake of processed red meat, including bacon, smoked ham, salami, frankfurter, Cumberland sausage, cold cuts and liver pâté. Total dietary fibers are calculated by the AOAC methods [51]. Pearson correlation coefficients (adjusted for total energy intake) illustrating the comparison of nutient scores estimated from the food-frequency questionnaire and from the diet records were 0.39 and 0.53 for dietary fibers, 0.56 and 0.48 for iron, and 0.37 and 0.14 for meat for men and women, respectively [52,53]. Alcohol intake was recorded as the average frequency of intake of six types of alcoholic beverage over the preceding year: the frequency of consumption of three types of beer was recorded in bottles (330 ml), wine in glasses (125 ml), and fortified wine in drinks (60 ml) and spirits in drinks (30 ml). The predefined responses were in 12 categories, ranging from "never" to "8 or more times a day". The alcohol content was calculated as follows: one bottle of light beer, 8.9 g ethanol; one bottle of regular beer, 12.2 g ethanol; one bottle of strong beer, 17.5 g ethanol; one glass of wine, 12.2 g ethanol; one drink of fortified wine, 9.3 g ethanol; and one drink of spirits, 9.9 g ethanol. We did not differentiate between red and white wine. Smoking intensity was calculated as gram tobacco smoked per day and included information on cigarettes (one cigarette = 1 g tobacco), cigars (one cigar = 4.5 g tobacco), cheroot (one cheroot = 4 g tobacco), and pipe (one pipe = 3 g tobacco).

The lifestyle questionnaire included this question regarding use of NSAID: "Have you taken more than one pain relieving pill per month during the last year?" If the answer was yes, the participant was asked to record how frequently they took each of the following medications: "Aspirin", "Paracetamol", "Ibuprofen", or "Other pain relievers". The latter category included NSAID preparations other than aspirin and ibuprofen. Based on all records, we classified study subjects according to use of "any NSAID" (≥ 2 pills per month during one year) at baseline.

Data on hormone replacement therapy (HRT), intake of dietary fibre and red meat, and anthropometric measurements was obtained as previously described [49]. The body mass index (BMI) was calculated as weight (kg) per height (m) squared.

### Blood sampling and storage

Blood was collected at enrolment and prepared as previously described [54]. In short, a total of 30 ml blood was collected in citrated (2 × 10 ml) and plain (1 × 10 ml) Venojects from each non-fasting participant. Plasma, serum, lymphocytes, and erythrocytes were isolated and frozen at -20°C within 2 hours. At the end of the day of collection, all samples were stored in liquid nitrogen, at -150°C.

### Genotyping

All analyses were run blinded to the case-control status. DNA was isolated from frozen lymphocytes as described by Miller et al [55]. Generally, 100 μg DNA were obtained from 10^7 ^lymphocytes. Genotyping was performed by TaqMan real-time quantitative PCR (QPCR).

*MDR1 *C3435T and G-rs3789243-A, *BCRP *C421A, and *COX-2 *G-765C were genotyped on an Mx3000 machine (Stratagene, La Jolla, CA, USA), using the Allelic Discrimination feature of the MxPro software (Stratagene). Reactions were carried out essentially as previously described [30], except the reaction volume was 15 μl. In brief, each reaction contained 1× TaqMan Universal Master Mix (Applied Biosystems, Foster City, CA, USA), approximately 20 ng DNA, and the relevant sets of primers and locked nucleic acid (LNA)-containing probes. All reactions were run for 50 cycles with two PCR steps, denaturation and combined annealing and elongation, respectively, except for *MDR1 *G-rs3789243-A, where annealing and elongation were split. Verified genotype controls were included in each run. To confirm reproducibility, 20 samples for each SNP were randomly selected within each of the three genotype groups and repeated. The genotypes showed 100% identity.

*COX-2 *A-1195G and T8473C were genotyped on an ABI7500 machine by endpoint readings as previously described [54]. Twenty ng of DNA were genotyped in 5 μl containing 1× Mastermix (Applied Biosystems, Nærum, Denmark), 100 nM probes, and 900 nM primers. Controls were included in each run, and repeated genotyping of a random 10% subset yielded 100% identical genotypes.

### Statistical Analysis

The analyses were performed according to the principles for the analysis of case-cohort studies as described by Barlow [56]. The analyses were performed unweighted. Age was used as the time scale in the Cox regression model. Tests and confidence intervals were based on Wald's test using the robust estimate of the variance-covariance matrix for the regression parameters in the Cox regression model [57].

All models were adjusted for baseline values of established risk factors for colorectal cancer such as BMI (kg/m^2^, continuous), NSAID (yes/no), use of HRT (never/past/current, among women), smoking status (never/past/current), and intake of dietary fibers (g/day, continuous) and red meat (g/day, continuous).

We investigated possible interactions between the genes and selected environmental factors using the likelihood ratio test. Trend test were calculated using the Wald test. The procedure PHREG in SAS (release 9.1; SAS Institute Inc., Cary, NC, USA) was used for the statistical analyses.

### Ethics

All participants gave verbal and written informed consent. Diet, Cancer and Health and the present sub-study were approved by the regional Ethics Committees on Human Studies in Copenhagen and Aarhus (Jr.nr. (KF)11-037/01 and jr.nr. (KF)01-045/93), and by the Danish Data Protection Agency.

## Results

### Associations between genotypes and CRC risk

Characteristics of the study population and risk factors for CRC are shown in Table 1.

**Table 1:** Baseline characteristics of study participants selected from the Danish Diet, Cancer and Health prospective cohort study.

	Cases		Sub-cohort		IRR^a ^(95% CI)	
	**No. (%)**	**Median (5-95%)**	**No. (%)**	**Median (5-95%)**		
Total	359 (100)		765 (100)			
						
Gender						
Men	200 (56)		419 (55)			
Women	159 (44)		346 (45)			
						
Age at inclusion		59 (51-64)		56 (50-64)		
						
Topology						
Proximal segment of colon	42 (12)		-			
Distal segment of colon	142 (40)		-			
Rectal	129 (36)		-			
Not specified	46 (13)		-			
						
BMI, kg/m^2^		26 (21-34)		26 (20-33)	1.02^b^	(0.96-1.09)
						
Food intake						
Alcohol, g/day		14 (1-69)		13 (1-64)	1.06^b^	(1.00-1.13)
Red meat, g/day		82 (36-170)		82 (32-175)	1.02^b^	(0.94-1.12)
Processed meat, g/day		26 (6-80)		26 (4-78)	1.00^b^	(0.85-1.19)
Dietary fibers g/day		20 (10-32)		21 (11-34)	0.62^b^	(0.37-1.02)
						
Smoking status at inclusion						
Never	111 (31)		262 (34)		1.00	-
Former	112 (31)		239 (31)		1.02	(0.73-1.42)
Present	136 (38)		264 (35)		1.15	(0.83-1.61)
						
NSAID use						
No	244 (68)		528 (69)		1.00	-
Yes	115 (32)		237 (31)		1.07	(0.80-1.42)
						
HRT use among women						
Never	93 (58)		181 (52)		1.00	-
Former	25 (16)		60 (17)		0.68	(0.39-1.18)
Present	41 (26)		105 (30)		0.70	(0.44-1.10)

^a ^Mutually adjusted.

^b ^BMI pr. 2 kg/m^2^. Alcohol pr. 10 g/day. Red meat, processed meat and dietary fibers pr 25 g/day.

Observed median values (5-95 percentiles) or percents of the distribution of alcohol, NSAID, smoking and potential colorectal cancer confounders among cases and members of the comparison group.

The genotype distributions among the participants in the sub-cohort sample did not deviate from Hardy-Weinberg equilibrium (results not shown). Carriers of the variant allele of *MDR1 *G-rs3789243-A were at 1.52-fold (95% confidence interval (CI): 1.12-2.06) higher risk of CRC than homozygous carriers of the wild type allele (Table 2). Carriers of the variant allele of *MDR1 *C3435T exon 26 had a lower risk of CRC than homozygous C-allele carriers (IRR = 0.71 (CI: 0.50-1.00)). *COX-2 *and *BRCP *polymorphisms were not associated with CRC risk (Table 2).

**Table 2:** Incidence rate ratio for colorectal cancer for the studied gene polymorphisms.

	N_Case_	N_Sub-cohort_	IRR^a^	(95% CI)	IRR^b^	(95% CI)	P-value^c^
*MDR1 *G-rs3789243-A							
GG	81	224	1.00	-	1.00	-	0.03
GA	194	365	1.55	(1.13-2.12)	1.55	(1.12-2.14)	
AA	84	176	1.43	(0.98-2.08)	1.45	(0.99-2.13)	
GA and AA	278	541	1.51	(1.12-2.04)	1.52	(1.12-2.06)	
							
*MDR1 *C3435T							
CC	73	118	1.00	-	1.00	-	0.11
CT	174	385	0.69	(0.49-0.99)	0.74	(0.51-1.07)	
TT	112	262	0.66	(0.45-0.96)	0.66	(0.45-0.98)	
CT and TT	286	647	0.68	(0.48-0.95)	0.71	(0.50-1.00)	
							
*COX-2 *G-765C							
GG	267	566	1.00	-	1.00	-	0.91
CG	83	186	1.02	(0.75-1.39)	1.03	(0.75-1.41)	
CC	9	13	1.45	(0.61-3.48)	1.21	(0.49-2.98)	
CG and CC	92	199	1.05	(0.78-1.41)	1.04	(0.77-1.42)	
							
*COX-2 *A-1195G							
AA	230	482	1.00	-	1.00	-	0.88
AG	116	258	0.94	(0.71-1.24)	0.93	(0.70-1.23)	
GG	13	25	0.90	(0.44-1.85)	0.94	(0.45-1.95)	
AG and GG	129	283	0.93	(0.71-1.22)	0.93	(0.71-1.23)	
							
*COX-2 *T8473C							
TT	147	315	1.00	-	1.00	-	0.37
CT	178	355	1.12	(0.85-1.48)	1.11	(0.83-1.47)	
CC	34	95	0.82	(0.52-1.29)	0.81	(0.51-1.28)	
CT and CC	212	450	1.06	(0.81-1.38)	1.05	(0.80-1.37)	
							
*BRCP *C421A							
CC	296	592	1.00	-	1.00	-	0.16
CA	58	161	0.72	(0.51-1.01)	0.71	(0.50-1.01)	
AA	5	12	1.10	(0.38-3.21)	1.17	(0.39-3.57)	
CA and AA	63	173	0.74	(0.53-1.03)	0.73	(0.52-1.04)	

^a^Crude (adjusted for age and sex).

^b^Adjusted for status of HRT (women only), smoking status, alcohol, dietary fibre, red meat, BMI and NSAID.

^c^p-value for trend for the fully adjusted estimates.

### Haplotype analyses

The two *MDR1 *polymorphisms were in incomplete linkage such that the two variant alleles co-segregated. Homozygous carriers of the combination of the C-allele of *MDR1 *C3435T and the A-allele of *MDR1 *G-rs3789243-A were at 1.80 fold higher risk of CRC (95% CI: 1.06-3.05) than homozygous carriers of the combination of the T-allele of *MDR1 *C3435T and the G-allele of *MDR1 *G-rs3789243-A. Carrier of only one of the risk alleles were generally also at higher risk of cancer, indicating that both polymorphisms contributed to the association with risk of CRC (Table 3).

**Table 3:** Incidence rate ratio for colorectal cancer for combinations of *MDR1 *genotypes

	IRR^a ^(95% CI) (N_case_/N_sub-cohort_)					
	***MDR1 *C3435T**					
***MDR1 *G-rs3789243-A**	**CC**		**CT**		**TT**	
GG	1.38	(0.31-6.15)	1.16	(0.66-2.06)	1.00^b^	-
N_case_/N_sub-cohort_	(3/6)		(27/68)		(52/153)	
GA	2.20	(1.22-3.96)	1.52	(1.01-2.29)	1.72	(1.06-2.81)
N_case_/N_sub-cohort_	(32/42)		(110/234)		(55/92)	
AA	1.80	(1.06-3.05)	1.38	(0.82-2.32)	1.48	(0.58-3.75)
N_case_/N_sub-cohort_	(39/72)		(38/85)		(8/20)	

^a ^Adjusted for status of HRT, smoking status, alcohol, dietary fibre, red meat and BMI.

^b^The most prevalent double homozygous genotype was used as reference.

Haplotype analyses combining the three polymorphisms of *COX-2 *revealed that four haplotypes included 97% of the observed genotype combinations in the cohort sample. *COX-2 *haplotypes were not associated with CRC risk (results not shown).

### Gene-gene and gene-environment analyses

Since we observed no gene-dose effects, variant genotypes were combined in subsequent interaction analyses to maximize the statistical power. No gene-gene interaction between the *MDR1 *G-rs3789243-A and *COX-2 *T8473C polymorphisms was found (results not shown). Since we observed no allele-dose effects, variant genotypes were combined in subsequent interaction analyses to maximize the statistical power. There was interaction between the studied *MDR1 *polymorphisms and intake of red and processed meat in relation to CRC risk (Table 4). Variant allele carriers of *MDR1 *G-rs3789243-A were at 3% higher risk pr 25 g meat/day (CI: 0.98-1.09) whereas for homozygous carriers of the wild type allele the association was in the opposite direction although not statistically significant (IRR pr 25 g/day: 0.95, CI: 0.89-1.02, p for interaction = 0.01). Homozygous *MDR1 *C3435T C-allele carriers were at 8% higher risk pr 25 g meat/day (CI: 1.00-1.16) whereas variant allele carriers were not at risk by meat intake (IRR pr 25 g/day 1.00, CI: 0.95-1.06, p for interaction = 0.02). No interaction was found for *BCRP *C421A.

**Table 4:** Interaction between intake of red and processed meat and *MDR1 *and *BCRP *polymorphisms in relation to CRC risk.

	N_Case_	N_Sub-cohort_	IRR^a^	(95% CI)	IRR^b^	(95% CI)	P-value^c^
*MDR1 *G-rs3789243-A							
GG	81	224	0.95	(0.89-1.02)	0.95	(0.89-1.02)	0.01
GA and AA	278	541	1.03	(0.98-1.08)	1.03	(0.98-1.09)	
							
*MDR1 *C3435T							
CC	73	118	1.07	(1.00-1.15)	1.08	(1.00-1.16)	0.02
CT and TT	286	647	1.00	(0.94-1.05)	1.00	(0.95-1.06)	
							
*BRCP *C421A							
CC	296	592	1.02	(0.97-1.07)	1.02	(0.97-1.08)	0.40
CA and AA	63	173	0.99	(0.91-1.07)	0.99	(0.91-1.08)	

IRR for colorectal cancer pr intake of additionally 25 g red or processed meat in relation to risk of CRC.

^a^Crude (adjusted for age and sex).

^b^Adjusted for status of HRT (women only), smoking status, alcohol, dietary fibre, BMI and NSAID.

^c^p-value for interaction between genotype and red and processed meat for the fully adjusted estimates.

We found interaction between *MDR1 *C3435T and NSAID use in relation to risk of CRC (Table 5). Among homozygous carriers of the C-allele, NSAID use was associated with 2.34-fold (CI: 1.22-4.48) higher risk of CRC compared to non-users, whereas NSAID use had no effect among variant allele carriers (p for interaction 0.001). Likewise, there was a marginally statistically significant interaction between *COX-2 *T8473C and NSAID use (p for interaction 0.04). A statistically non-significantly increased risk of CRC by NSAID use was found among homozygous wild type allele carriers (IRR 1.38, CI: 0.89-2.14) compared to non-NSAID users, whereas NSAID use was not associated with increased CRC risk among variant allele carriers (Table 5). The same trend was seen for the *COX-2 *G-765C polymorphism (p = 0.06).

**Table 5:** Interactions between nonsteroidal anti-inflammatory drug (NSAID) use and *MDR1, COX-2 *and *BCRP *polymorphisms.

Polymorphism	N_case_/N_sub-cohort_		IRR^a ^(95% CI)				IRR^b ^(95% CI)				p-value^c^
	NSAID^d^		NSAID^d^				NSAID^d^				
	NO	YES	NO		YES		NO		YES		
*MDR1 *C3435T											
CC	41/79	32/39	1.00	-	2.21	(1.17-4.17)	1.00	-	2.34	(1.22-4.48)	0.001
CT and TT	204/449	82/198	0.92	(0.60-1.41)	0.83	(0.52-1.33)	0.99	(0.63-1.54)	0.86	(0.53-1.39)	
											
*MDR1 *G-rs 3789243-A											
GG	54/160	27/64	1.00	-	1.35	(0.77-2.35)	1.00	-	1.31	(0.74-2.32)	0.26
GA and AA	191/368	87/173	1.65	(1.15-2.38)	1.65	(1.09-2.49)	1.66	(1.15-2.41)	1.61	(1.06-2.46)	
											
*COX-2 *G-765C											
GG	180/397	87/169	1.00	-	1.22	(0.89-1.69)	1.00	-	1.20	(0.86-1.67)	0.06
CG and CC	65/131	27/68	1.22	(0.86-1.75)	0.93	(0.57-1.51)	1.23	(0.85-1.78)	0.90	(0.54-1.48)	
											
*COX-2 *A-1195G											
AA	156/329	74/153	1.00	-	1.05	(0.75-1.48)	1.00	-	0.99	(0.69-1.41)	0.46
AG and GG	89/199	40/84	0.91	(0.66-1.27)	1.04	(0.67-1.60)	0.88	(0.63-1.23)	1.04	(0.67-1.61)	
											
*COX-2 *T8473C											
TT	94/222	53/93	1.00	-	1.42	(0.92-2.18)	1.00	-	1.38	(0.89-2.14)	0.04
CT and CC	151/306	61/144	1.23	(0.89-1.70)	1.09	(0.73-1.62)	1.22	(0.87-1.69)	1.05	(0.70-1.58)	
											
*BCRP *C421A											
CC	199/414	97/178	1.00	-	1.20	(0.88-1.63)	1.00	-	1.18	(0.85-1.62)	0.09
CA and AA	46/114	17/59	0.86	(0.58-1.27)	0.62	(0.35-1.10)	0.86	(0.57-1.29)	0.60	(0.33-1.08)	

^a^Crude (adjusted for age and sex).

^b^Adjusted for status of HRT (women only), smoking status, alcohol, dietary fibre, red meat and BMI.

^c^p-value for interaction for the fully adjusted risk estimates.

^d^Study subjects were classified according to use of "any NSAID" (≥ 2 pills per month during one year) at baseline

There was no interaction between the polymorphisms and smoking status in relation to CRC risk (results not shown).

## Discussion

In the present study, both of the studied *MDR1 *polymorphisms were associated with CRC risk and interacted with meat intake in relation to CRC risk. Carriers of the *MDR1 *G-rs3789243-A A-allele and homozygous carriers of *MDR1 *C3435T C-allele were at higher risk of CRC than carriers of the common allele genotypes. Carriers of these two genotypes were, moreover, at higher risk of CRC in relation to intake of red and processed meat whereas carriers of the respective common allele genotypes were not at higher risk of CRC in relation to meat intake. *COX-2 *and *BCRP *gene polymorphisms were not associated with CRC risk. Furthermore, there was interaction between NSAID use and the *MDR1 *C3435T and *COX-2 *T8473C polymorphisms in relation to risk of CRC. No gene-smoking interactions were found.

The present study design has pros and cons. Prospective studies have the advantage in relation to examining gene-environmental interactions that they are not encumbered by recall bias. In the present study, cases and cohort sample were selected from the same cohort, which together with complete follow up of the participants, minimised the risk of selection bias. Information on lifestyle factors were collected at enrolment for all participants which minimised the risk of differential misclassification of cases and comparison group. However, lifestyle factors were only collected once, and may thus not be representative for the lifestyle during follow-up. This is, however, not expected to result in differential misclassification. Furthermore, information on food intake was based on a semi-quantitatively food frequency questionnaire [49,53], which was, however, evaluated and found useable [52].

Known life-style factors affecting the risk of CRC include diet, physical activity, body mass index (BMI), alcohol, smoking and NSAID use [15]. In this study the results were adjusted for relevant confounding factors. Physical activity (habitual exercise) has been shown not to be a risk factor in this population and hence, was not adjusted for [58].

Heterozygous and homozygous variant genotype carriers were combined for the analyses of interactions due to power-considerations. Therefore, in the light of the obtained P-values and the number of statistical testes performed, we cannot exclude that our positive findings are due to chance. On the other hand, the fact that we found interaction between both of the studied *MDR1 *polymorphisms and meat intake in relation to CRC risk makes a chance finding less likely.

In contrast to the finding in the present study, *MDR1 *polymorphisms have not been associated with overall risk of CRC in previous studies [31-33]. The only other larger study, a case-cohort study in a Norwegian population of the polymorphism G-rs3789243A [29,30] found no association between this SNP and the development of intestinal adenomas and carcinomas [3]. Smaller studies, including up to 285 cases, have not found associations with overall CRC risk among Caucasians or Koreans [31-33], although an association was found between the *MDR1 *C3435T variant allele and CRC risk among patients diagnosed before the age of 50 years [31]. Moreover, we found no interaction between the *MDR1 *polymorphism and smoking status which is in contrast to a previous study showing an association between *MDR1 *C3435T variant allele and CRC risk among life-long nonsmokers of more than 63 years of age [33]. Relative to our study, the mentioned studies have a weaker design.

We found a relative risk by meat intake of 1.03 (95% CI: 0.98-1.09, p for interaction = 0.01) and 1.08 (95% CI: 1.00-1.16, p for interaction 0.02) per 25 g red and processed meat per day for the two identified risk *MDR1 *genotypes. The risk is in line with a previous finding of a relative risk of 1.29 per 100 g per day [16]. Moreover, meat intake was not associated with CRC risk for carriers of the other genotypes. The found interactions between *MDR1 *polymorphisms and intake of meat in the present study suggest that the *MDR1 *polymorphisms may be of minor importance in study populations with a low intake of red and processed meat.

We observed an interaction between *MDR1 *C3435T and NSAID use such that NSAID use was associated with increased CRC risk among homozygous carriers of the wild type allele only. In cell lines, COX-2 has been shown to induce P-glycoprotein [59] whereas COX-2 inhibition prevented the expression and function of P-glycoprotein [60], thereby affecting apoptosis [61]. A similar relationship has been found *in vivo *[62]. Thus, NSAID use seems to affect P-glycoprotein tumorigenesis in an incompletely understood manner. While *MDR1 *seems to play a role in CRC carcinogenesis, much is to be learned about the function of P-glycoprotein in relation to carcinogenesis.

The silent *MDR1 *C3435T polymorphism has been suggested to lead to a more unstable mRNA and consequently, lower overall activity of the variant allele should be expected [24]. Therefore, we expected that red and processed meat would be most carcinogenic among T-allele carriers of *MDR1 *C3435T, whereas the opposite was found. However, in a very recent review, Fung et al suggest that the silent C3435 polymorphism induces a conformational change in P-glycoprotein due to ribosome stalling during translation, whereas no effect on mRNA stability was detected [25]. The two polymorphic P-glycoproteins were shown to differ in their substrate specificity, since transport of varapamil but not rapamycin was changed by the polymorphism [25]. Our study suggestthat red and processed meat were most carcinogenic among C-allele carriers of MDR1 C3435T. The very large *MDR1 *gene includes 28 exons and is highly polymorphic which makes it difficult to identify causal polymorphisms. In addition, linkage patterns and allele frequencies in *MDR1 *are highly variable between different ethnic groups and thus between the studied populations [22]. Hence, case-control studies with assessment of multiple polymorphisms, enabling comprehensive haplotype analysis, in parallel with P-glycoprotein activity, mRNA and protein level measurements, will be required to understand *MDR1 *genotype-phenotype causality.

We found no association between *BCRP *genotypes and risk of CRC and on interaction between *BCRP *genotypes and meat, smoking or NSAID. Our result is in accordance with a recent gene-wide association study [4]. Our study suggests that P-glycoprotein, but not BCRP, transport certain carcinogens, which are relevant in relation to meat intake. Therefore, elucidating substrate differences between P-glycoprotein and BCRP may help to identify possible mechanisms behind meat-related carcinogenesis [63].

We found no association between *COX-2 *genotypes and risk of CRC. The haplotype pattern was similar to what has previously been found for Danes [54]. In accordance with our results, no association between *COX*-2 polymorphisms and CRC was demonstrated in a large French case control study [64] and smaller studies [65,66]. Variant allele carriers of *COX-2 *G-765C have been reported to be at higher CRC risk among Han Chinese [42]. Other studies suggest that the effects of polymorphisms on *COX-2 *expression levels are large enough to have biological impact provided that *COX-2 *expression is important in CRC [54]. On this basis, our results suggest that COX-2 plays a limited role in colon carcinogenesis in the present study population.

Interaction between NSAID and *COX-2 *polymorphisms in relation to risk of CRC and colorectal adenomas has been investigated previously [65,67,68]. Although some studies on colorectal adenomas may suggest that the largest risk-reducing effect by NSAID use is observed among the genotypes related to high COX-2 levels, results are not consistent [68-70]. We observed interaction between *COX-2 *T8473C and NSAID use. NSAID use was associated with CRC risk among homozygous carriers of the COX-2 T8473C genotype, which is assumed to be associated with low expression levels. In accordance with our result, [45,61] aspirin has been shown to induce *COX-2 *transcription in some tissues, among others intestinal myofibroblasts, especially in the presence of IL-1β [71]. On the other hand, induction of *COX-2 *gene expression by inhibition of COX-2 have been demonstrated in both liver and colon cancer cell lines [45,61]. This seem to be mediated by a COX-2 independent mechanism [45], These in vitro results indicate that the tissue specific effects of NSAIDs are far from clear. Thus, the observed interaction may be a chance finding. In the 'Diet, Cancer and Health cohort, used in the present study, long term use of NSAID was associated with a protective effect against CRC [72]. However, using a higher intake of NSAID as cut-off value (weekly use) did not change our results regarding NSAID use (results not shown).

## Conclusion

In conclusion, the present study indicated that the two studied *MDR1 *polymorphisms are associated with risk of CRC and interact with intake of red and processed meat in relation to CRC risk, whereas no association was found between *BCRP *and *COX-2 *polymorphisms and risk of CRC. Our study suggests that genetic variations in *MDR1*, but not *BCRP*, affects the intestinal absorption of meat related dietary carcinogens and that elucidating substrate differences between P-glycoprotein and BCRP may identify possible mechanisms behind meat-related carcinogenesis. This is the first study finding interactions between polymorphisms affecting intestinal transport proteins and meat intake in relation to CRC.

Next, our results suggest an increased risk of CRC by NSAID intake by homozygote *MDR1 *C3435T wildtype carriers. If confirmed, the present results stresses the importance of identification of possible subgroups which may not take advantage of, or may even be at increased risk, by NSAID prevention. No consistent associations were observed for *COX-2 *polymorphisms.

## Abbreviations

*BCRP*: Breast Cancer Resistance Protein; BMI: body mass index; CI: confidence interval; *COX-2*: Cyclooxygenase-2; CRC: Colorectal Cancer; HRT: hormone replacement therapy; *MDR1*: Multidrug Resistance 1; IRR: incidence rate ratio; NSAID: non-steroidal anti-inflammatory drug; QPCR: real-time quantitative PCR; SNP: single nucleotide polymorphism.

## Competing interests

The authors declare that they have no competing interests.

## Authors' contributions

MØ and UV carried out the molecular genetic studies. UV, KO, AT participated in the design of the study and JC performed the statistical analysis. VA conceived the study, and participated in its design and coordination and VA, UV and JC drafted the manuscript. All authors read and approved the final manuscript.

## Pre-publication history

The pre-publication history for this paper can be accessed here:

http://www.biomedcentral.com/1471-2407/9/407/prepub
